# Supplementary material for: In silico identification, high yielding isolation and in vitro validation of 6β-cinnamoyl-7β -hydroxyvouacapen – 5α - ol as a Wnt/β-catenin pathway targeted anti-cancer secondary metabolite of Caesalpinia pulcherrima
Source: PLoS One. 2025 Nov 3;20(11):e0334238. doi: 10.1371/journal.pone.0334238 (PMC12582477; doi:10.1371/journal.pone.0334238)
Supplement: S1 Table — The secondary metabolites passing the drug-likeness filter (Lipinsik’s Rule of Five) were selected for further validation using molecular docking and molecular dynamics. L. (PDF) [file pone.0334238.s001.pdf]

Table S2. Drug likness evaluation of the secondary metabolites using SwissADME. The secondary metabolites passing the drug-likeness filter (Lipinsik's Rule of Five) were selected for further validation using molecular docking and molecular dynamics.

**Phytochemical Name**	**Binding Affinity**	**rmsd/lb**	**rmsd/lb**	**Drug likeness**
Myrtenol	-4.3	0	0	Pass
Bonducellin	-6.4	0	0	Pass
Flavylium	-5.8	0	0	Pass
Benzoic acid	-4.6	0	0	Pass
Myrcene	-3.9	0	0	Pass
Eugenol	-4.8	0	0	Pass
beta-Copaene	-5.3	0	0	Pass
4'-Hydroxy-5,6,7,8-tetramethoxyflavone	-5.7	0	0	Pass
Quercetin	-6.5	0	0	Pass
6-Methoxypulcherrimin	-7.2	0	0	Pass
alpha-Caesalpin	-6.2	0	0	Pass
Myricetin	-6.5	0	0	Pass
Ellagic acid	-6.6	0	0	Pass
(E)-7-hydroxy-8-methoxy-3-(4-methoxybenzylidene)chroman-4-one	-6.4	0	0	Pass
Spathulenol	-5.2	0	0	Pass
Pulcherrimin	-2.3	0	0	Pass
Ethyl gallate	-5.3	0	0	Pass
2-Nonanone	-3.7	0	0	Pass
Sebacic acid	-4.5	0	0	Pass
2,6-Dimethoxy-1,4-benzoquinone	-4.6	0	0	Pass
2-Undecanone	-3.8	0	0	Pass
(S,1Z,6Z)-8-Isopropyl-1-methyl-5-methylenecyclodeca-1,6-diene	-5.1	0	0	Pass
alpha-Muurolene	-5	0	0	Pass
Humulene epoxide II	-5.7	0	0	Pass
gamma-Muurolene	-4.9	0	0	Pass
(+)-gamma-Cadinene	-5	0	0	Pass
Leucodelphidin	-6.5	0	0	Pass
(+)-delta-Cadinene	-5.2	0	0	Pass
Gallic acid	-5.4	0	0	Pass
alpha-Pinene	-4.4	0	0	Pass

beta-Pinene	-4.2	0	0	Pass
alpha-Fenchol	-4	0	0	Pass
alpha-Terpineol	-4.6	0	0	Pass
Lupeol	-6.5	0	0	Pass
(-)-alpha-Cadinol	-5.1	0	0	Pass
Caryophyllene oxide	-5.5	0	0	Pass
Junenol	-5.4	0	0	Pass
epi-Cubenol	-5.2	0	0	Pass
(-)-Globulol	-5.3	0	0	Pass
Zizanene	-5.3	0	0	Pass
Aromadendrene	-5.3	0	0	Pass
beta-Sitosterol	-5.8	0	0	Pass
D-Glucose	-5.4	0	0	Pass
D-Fructose	-5.1	0	0	Pass
Limonene	-4.2	0	0	Pass
Lupeol acetate	-6.6	0	0	Pass
alpha-Muurolol	-5	0	0	Pass
Nerolidol	-4.8	0	0	Pass
D-Xylose	-4.9	0	0	Pass
alpha-Copaene	-5.1	0	0	Pass
T-Muurolol	-5.2	0	0	Pass
Allo-Aromadendrene	-5.1	0	0	Pass
(-)-trans-Pinocarveol	-4.4	0	0	Pass
Viridiflorol	-5.1	0	0	Pass
(-)-trans-Pinocarveol	-4.4	0	0	Pass
