# Supplementary material for: In silico identification, high yielding isolation and in vitro validation of 6β-cinnamoyl-7β -hydroxyvouacapen – 5α - ol as a Wnt/β-catenin pathway targeted anti-cancer secondary metabolite of Caesalpinia pulcherrima
Source: PLoS One. 2025 Nov 3;20(11):e0334238. doi: 10.1371/journal.pone.0334238 (PMC12582477; doi:10.1371/journal.pone.0334238)
Supplement: S2 Table — The scripts for MM-PBSA binding free energy calculations were adapted from publicly available websites. (PDF) [file pone.0334238.s002.pdf]

Table S1. Links to the GROMACS and binding free energy calculation scripts. GROMACS source code and associated tools were obtained form the official GROMACS website. The scripts for MM-PBSA binding free energy calculations were adapted from publicly available websites.

**Codes**	**Links**
GROMACS	https://tutorials.gromacs.org/md-intro-tutorial.html
Binding free energy calculation	https://valdes-tresanco-ms.github.io/gmx_MMPBSA/
