# Supplementary material for: In silico identification, high yielding isolation and in vitro validation of 6β-cinnamoyl-7β -hydroxyvouacapen – 5α - ol as a Wnt/β-catenin pathway targeted anti-cancer secondary metabolite of Caesalpinia pulcherrima
Source: PLoS One. 2025 Nov 3;20(11):e0334238. doi: 10.1371/journal.pone.0334238 (PMC12582477; doi:10.1371/journal.pone.0334238)
Supplement: S3 Table — The table summarizes the cytotoxic activity of the pooled fractions against NTERA-2 cells as a cancer stem cell (CSC) model. (PDF) [file pone.0334238.s003.pdf]

S3 Table. IC_{50} values of combined residual pooled fractions obtained from all the column fractions except fraction 2. The table summarizes the cytotoxic activity of the pooled fractions against NTERA-2 cells as a cancer stem cell (CSC) model.

**Fraction number**	**24h IC_{50} (µg/mL)**
**1**	>200
**2**	4.63
**3**	23.26
**4**	21.32
**5**	34.83
**6**	>200
**7**	>200
**8**	97.22
**9**	107.32
**10**	>200
**11**	>200
**12**	>200
