# Supplementary material for: In silico identification, high yielding isolation and in vitro validation of 6β-cinnamoyl-7β -hydroxyvouacapen – 5α - ol as a Wnt/β-catenin pathway targeted anti-cancer secondary metabolite of Caesalpinia pulcherrima
Source: PLoS One. 2025 Nov 3;20(11):e0334238. doi: 10.1371/journal.pone.0334238 (PMC12582477; doi:10.1371/journal.pone.0334238)
Supplement: S1 Fig — The figures illustrates the binding pose of the secondary metabolites in the binding pocket along with key molecular interactions hydrogen bonds, hydrophobic interactions and ionic interactions. (PDF) [file pone.0334238.s004.pdf]

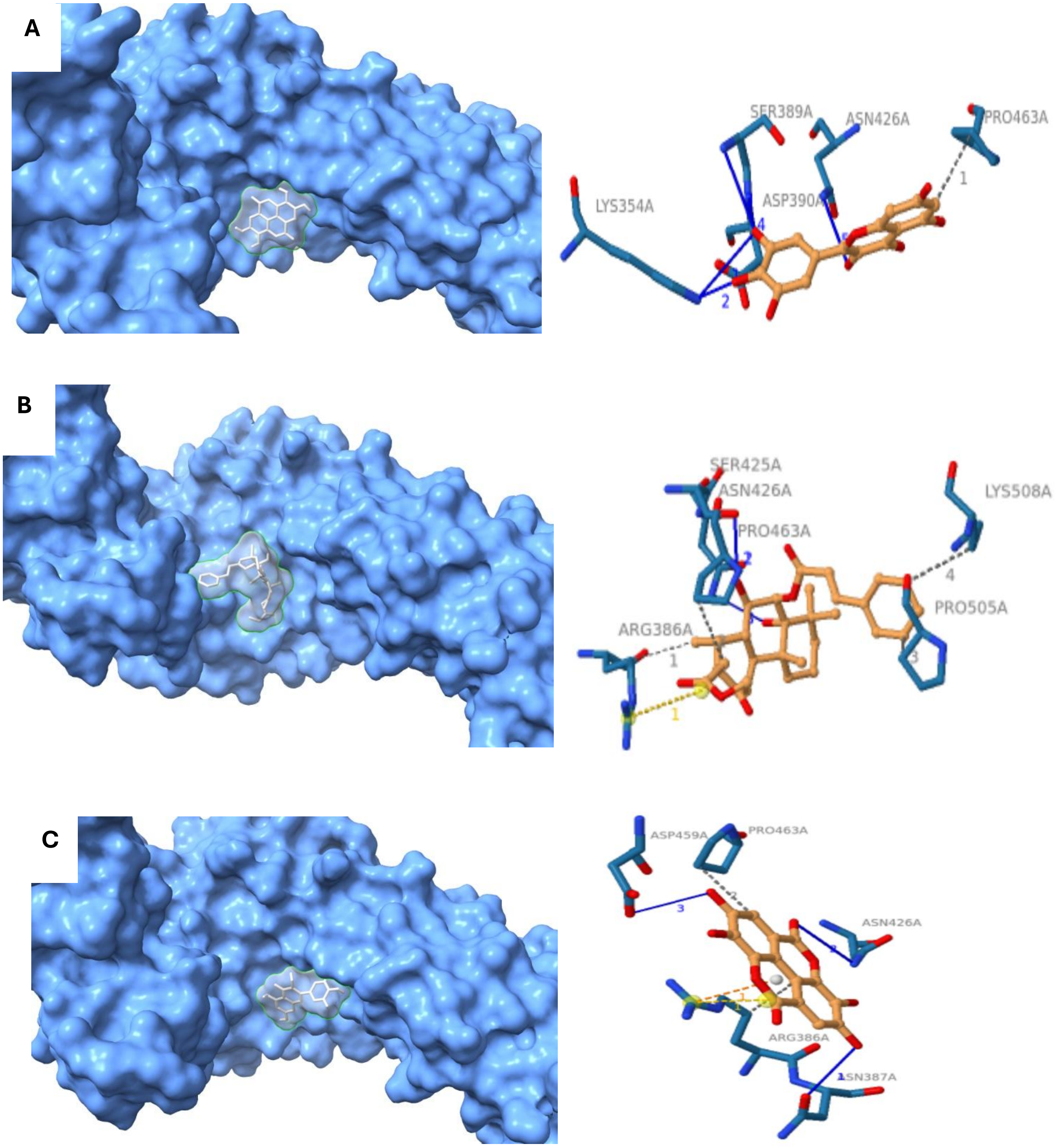
A
SER389A
ASN426A
PRO463A
LYS354A
ASP390A
B
SER425A
ASN426A
PRO463A
LYS508A
ARG386A
PRO505A
C
ASP459A
PRO463A
ASN426A
ARG386A
SN387A

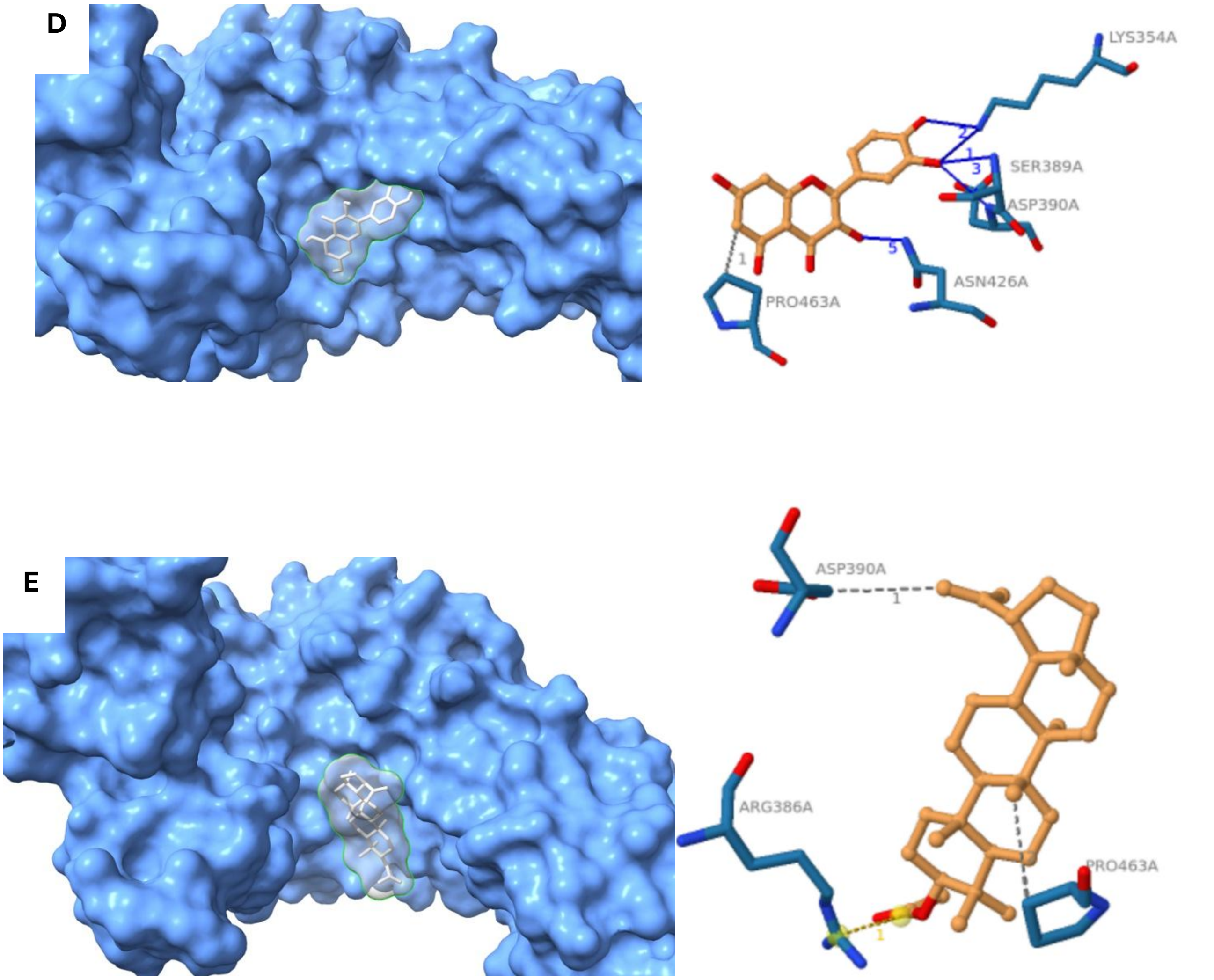

S1 Fig. Predicted binding poses of the selected ligand-protein complex. The figures illustrates the binding pose of the secondary metabolites in the binding pocket along with key molecular interactions hydrogen bonds, hydrophobic interactions and ionic interactions. (●Charged centre, ---- Hydrophobic interaction, —— Hydrogen bond , ···· Cation л interaction, ···· Salt bridge). A) Ellagic acid, B) Pulcherralpin, C) Myricetin, D) Quercetin, E) Lupeole Acetate.
