# Supplementary material for: In silico identification, high yielding isolation and in vitro validation of 6β-cinnamoyl-7β -hydroxyvouacapen – 5α - ol as a Wnt/β-catenin pathway targeted anti-cancer secondary metabolite of Caesalpinia pulcherrima
Source: PLoS One. 2025 Nov 3;20(11):e0334238. doi: 10.1371/journal.pone.0334238 (PMC12582477; doi:10.1371/journal.pone.0334238)
Supplement: S2 Fig — The graph illustrates the structural stability and conformational fluctuations of each ligand-protein complex throughout the simulation period of 200 ns. (PDF) [file pone.0334238.s005.pdf]

A

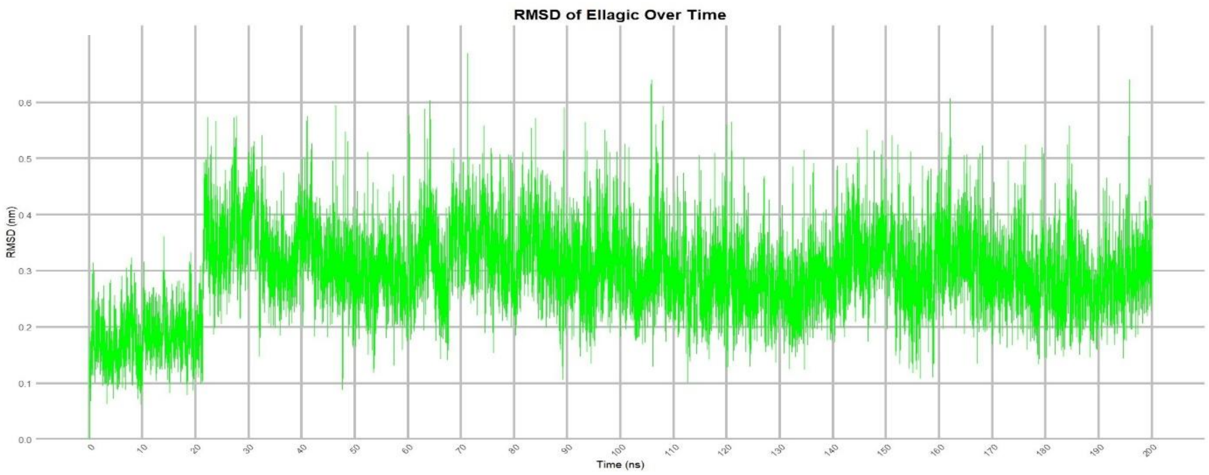
RMSD of Ellagic Over Time
RMSD (nm)
0.0
0.1
0.2
0.3
0.4
0.5
0.6
0
10
20
30
40
50
60
70
80
90
100
110
120
130
140
150
160
170
180
190
200
Time (ns)

B

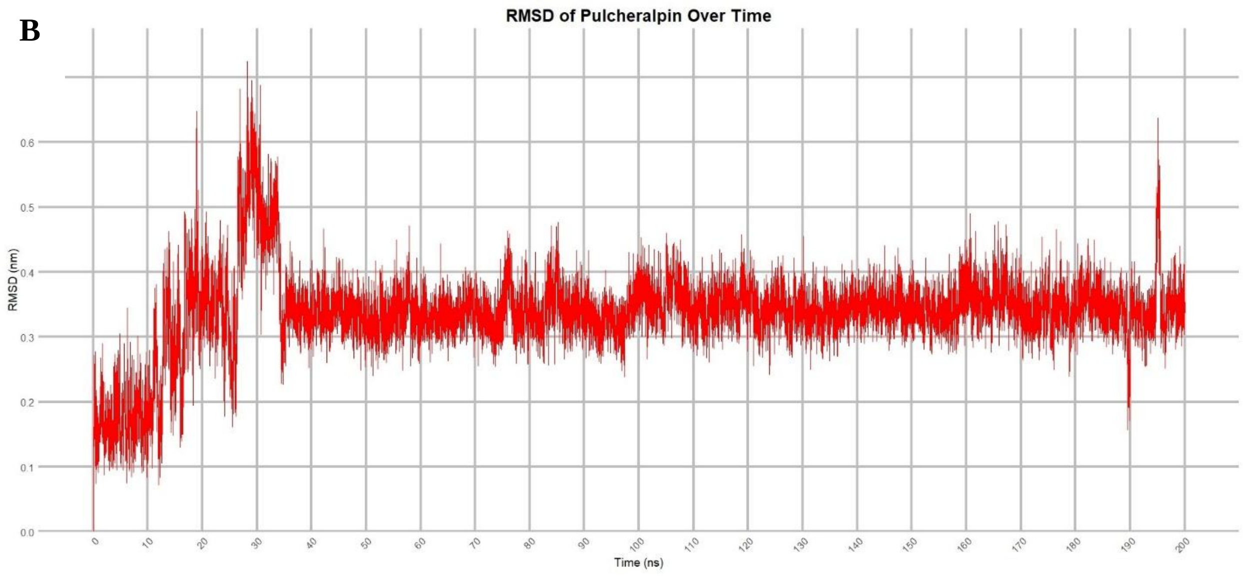
RMSD of Pulcheralpin Over Time
RMSD (nm)
0.0
0.1
0.2
0.3
0.4
0.5
0.6
0
10
20
30
40
50
60
70
80
90
100
110
120
130
140
150
160
170
180
190
200
Time (ns)

C

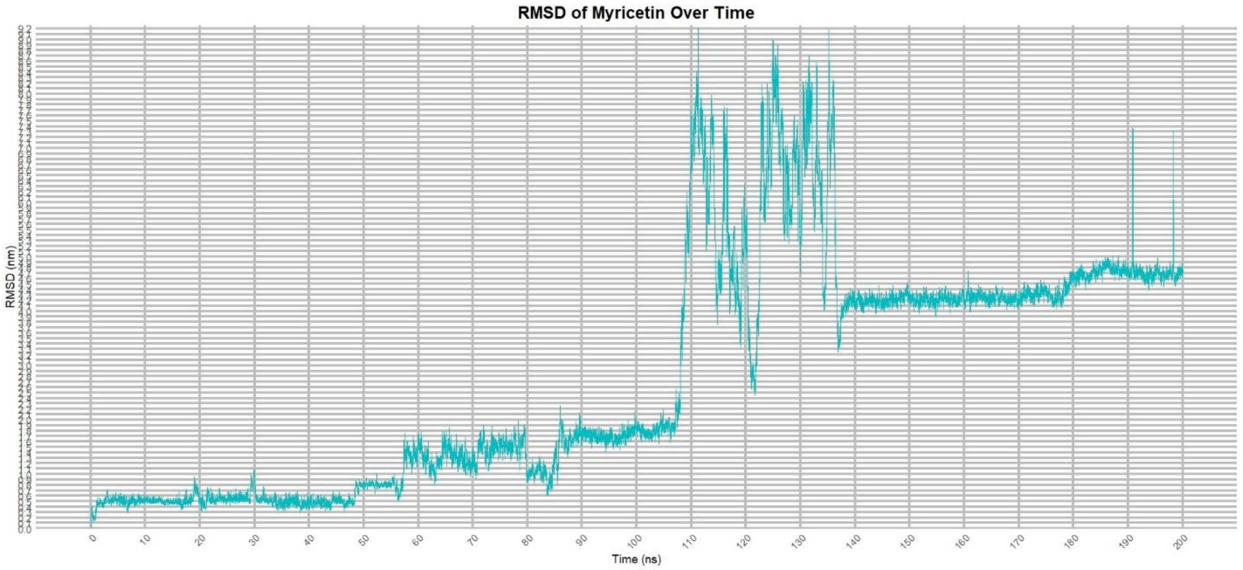
RMSD of Myricetin Over Time
RMSD (nm)
0 10 20 30 40 50 60 70 80 90 100 110 120 130 140 150 160 170 180 190 200
Time (ns)

D

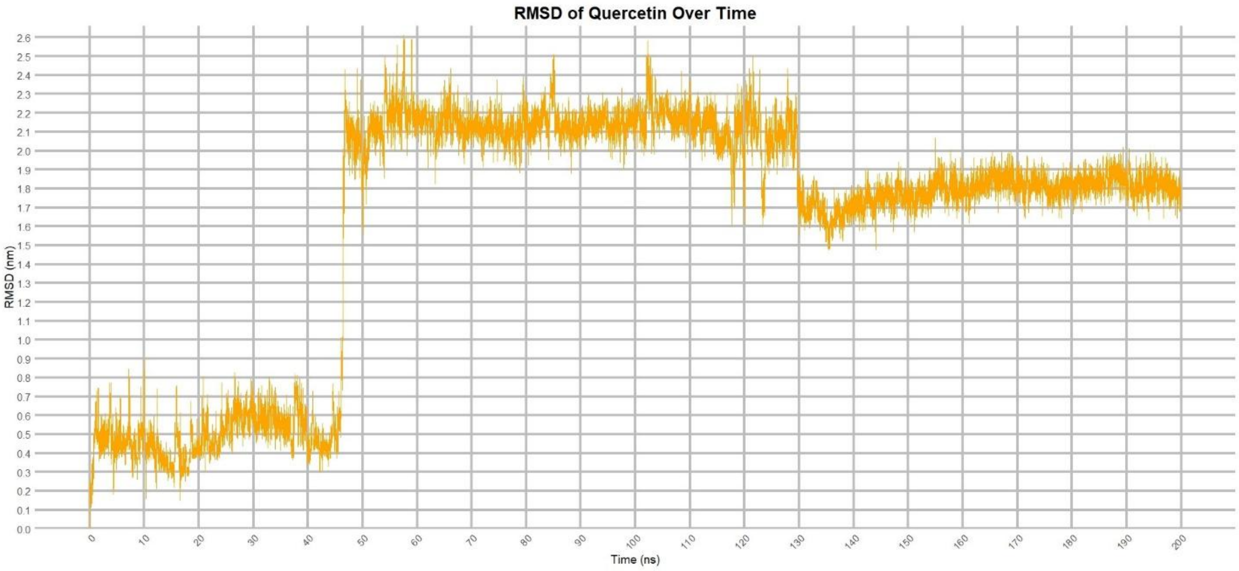
RMSD of Quercetin Over Time
RMSD (nm)
0.0 0.1 0.2 0.3 0.4 0.5 0.6 0.7 0.8 0.9 1.0 1.1 1.2 1.3 1.4 1.5 1.6 1.7 1.8 1.9 2.0 2.1 2.2 2.3 2.4 2.5 2.6
0 10 20 30 40 50 60 70 80 90 100 110 120 130 140 150 160 170 180 190 200
Time (ns)

E

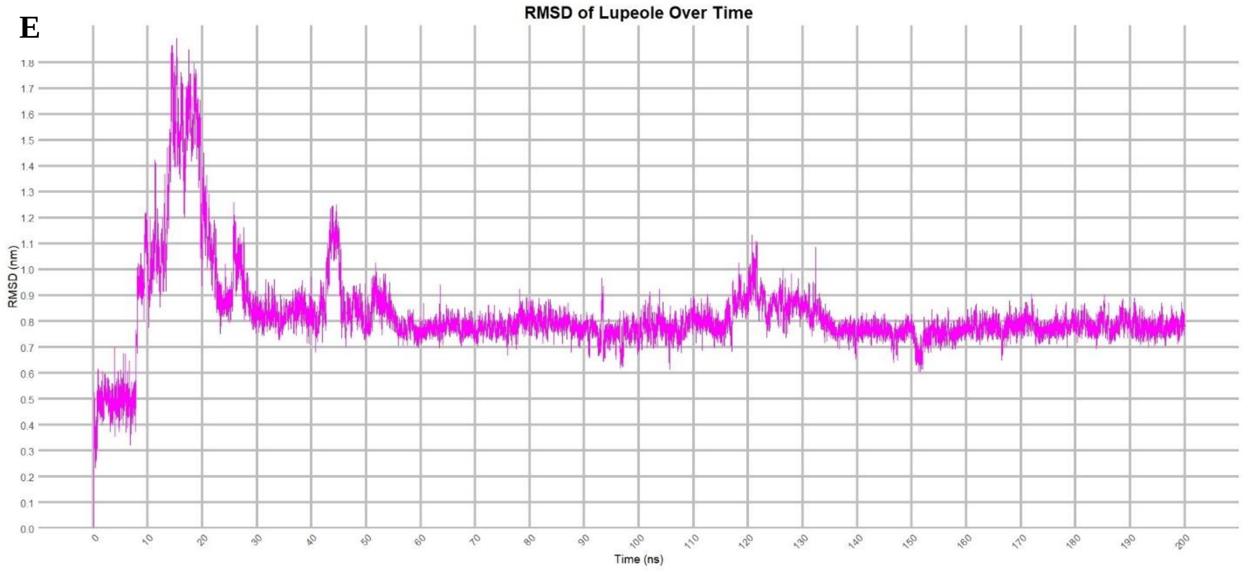

S2 Fig: Root Mean Square Deviation (RMSD) of secondary metabolites within the receptor binding site over a 200 ns simulation. The graph illustrates the structural stability and conformational fluctuations of each ligand-protein complex throughout the simulation period of 200ns. A) Ellagic acid, B) Pulcherralpin, C) Myricetin, D) Quercetin, E) Lupeole Acetate.
