# Supplementary material for: In silico identification, high yielding isolation and in vitro validation of 6β-cinnamoyl-7β -hydroxyvouacapen – 5α - ol as a Wnt/β-catenin pathway targeted anti-cancer secondary metabolite of Caesalpinia pulcherrima
Source: PLoS One. 2025 Nov 3;20(11):e0334238. doi: 10.1371/journal.pone.0334238 (PMC12582477; doi:10.1371/journal.pone.0334238)
Supplement: S3 Fig — The plot demonstrate the flexibility of amino acid residues at the binding site, providing details into conformational changes and interaction stability throughout the simulation of 200 ns. (PDF) [file pone.0334238.s006.pdf]

A

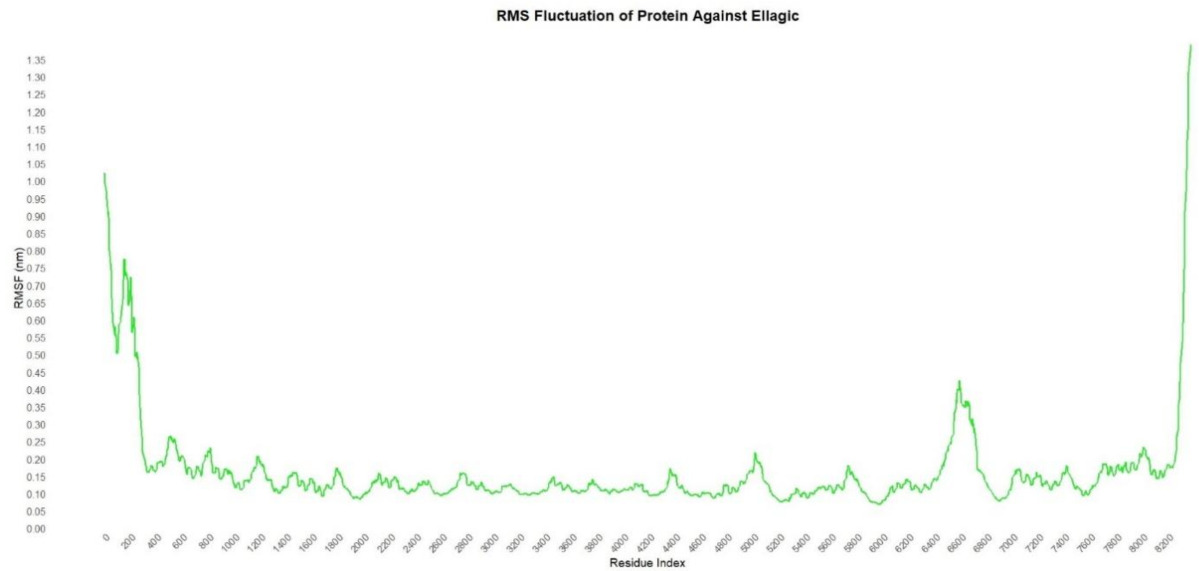
RMS Fluctuation of Protein Against Ellagic
RMSF (nm)
Residue Index

B

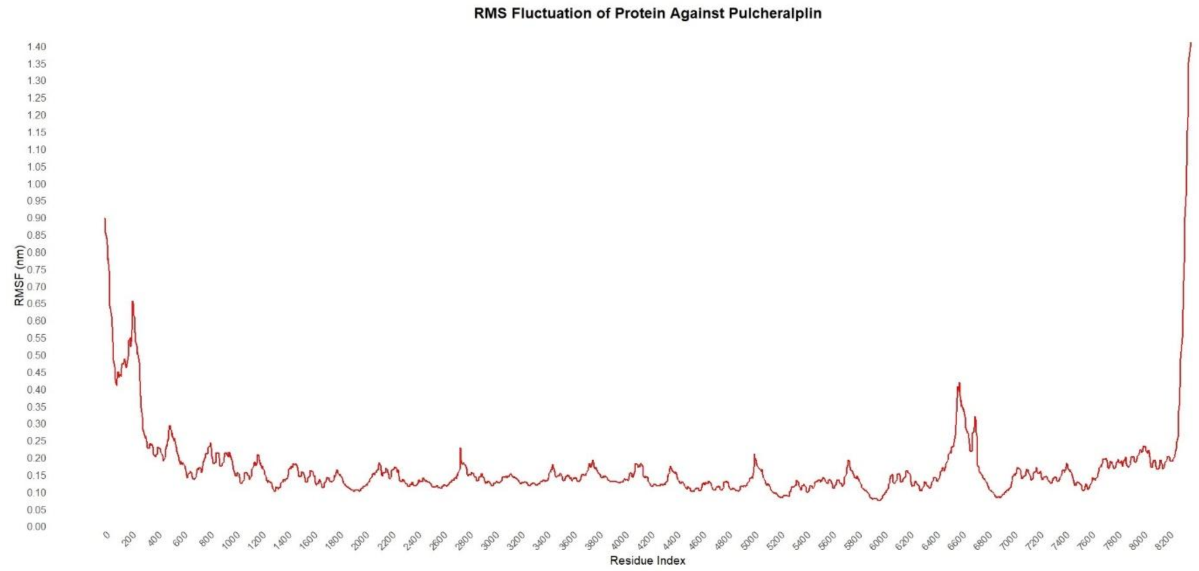
RMS Fluctuation of Protein Against Pulcheralplin
RMSF (nm)
Residue Index

C

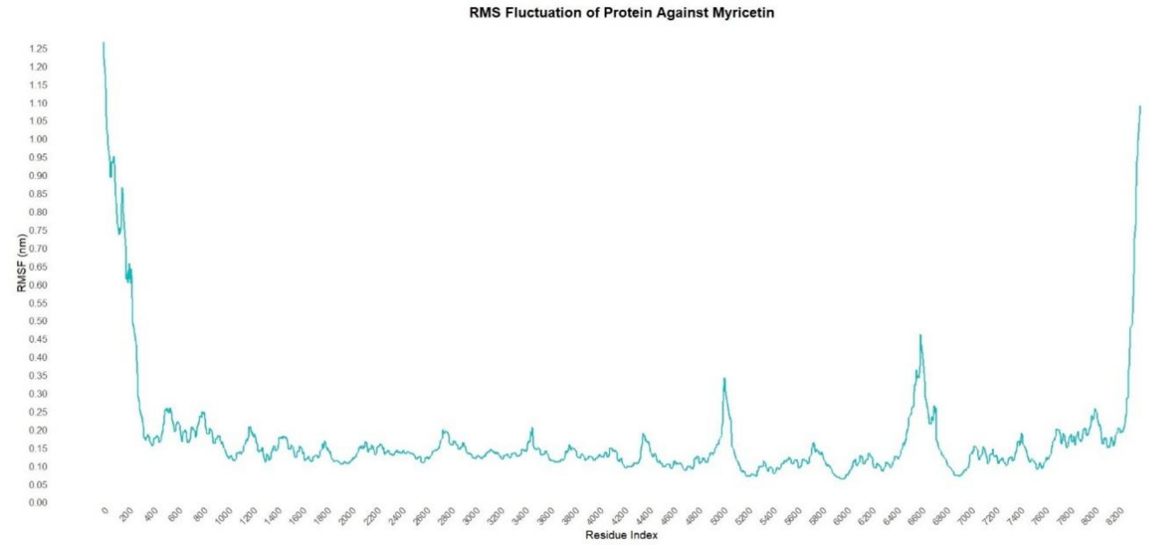
RMS Fluctuation of Protein Against Myricetin
RMSF (nm)
Residue Index

D

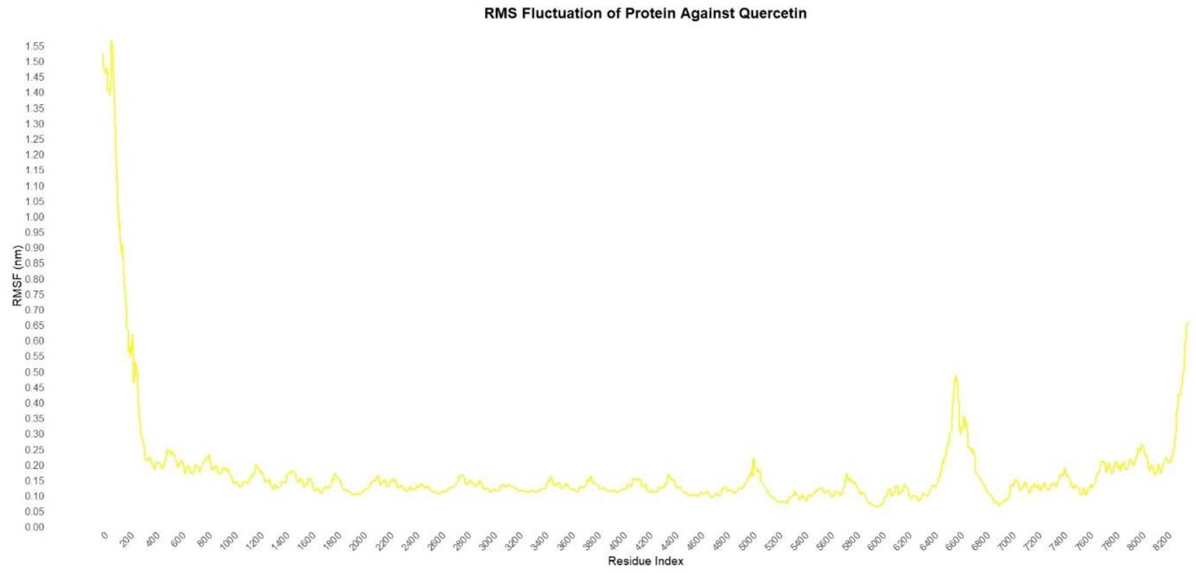

E

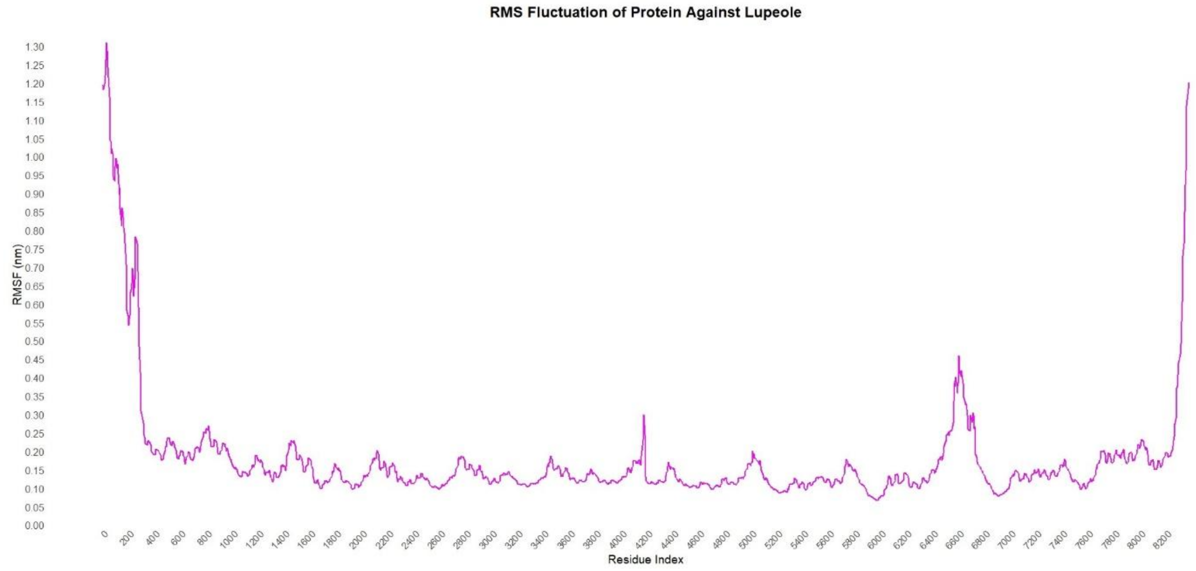

S3 Fig: Root Mean Square Fluctuation (RMSF) of the receptor residues interacting with secondary metabolites over a 200 ns simulation. The plot demonstrate the flexibility of amino acid residues at the binding site, providing details into conformational changes and interaction stability throughout the simulation of 200ns. A) Ellagic acid, B) Pulcherralpin, C) Myricetin, D) Quercetin, E) Lupeole Acetate.
