# Supplementary material for: In silico identification, high yielding isolation and in vitro validation of 6β-cinnamoyl-7β -hydroxyvouacapen – 5α - ol as a Wnt/β-catenin pathway targeted anti-cancer secondary metabolite of Caesalpinia pulcherrima
Source: PLoS One. 2025 Nov 3;20(11):e0334238. doi: 10.1371/journal.pone.0334238 (PMC12582477; doi:10.1371/journal.pone.0334238)
Supplement: S4 Fig — The figure indicate the stability and strength of the ligand-protein interactions. (PDF) [file pone.0334238.s007.pdf]

A

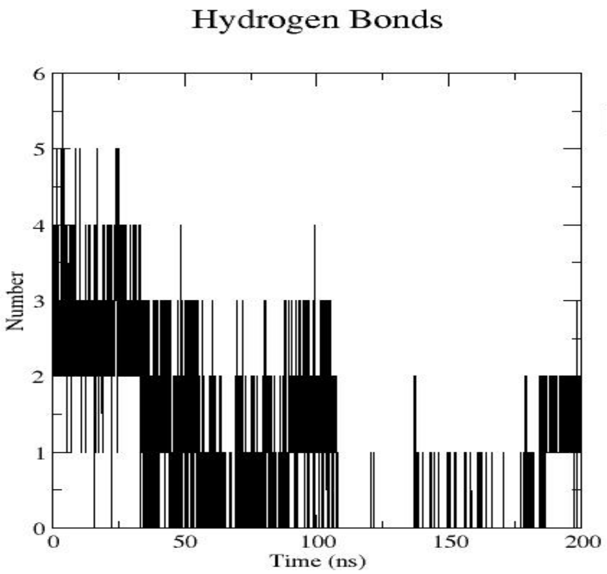
Hydrogen Bonds
Number
0
1
2
3
4
5
6
Time (ns)
0
50
100
150
200

B

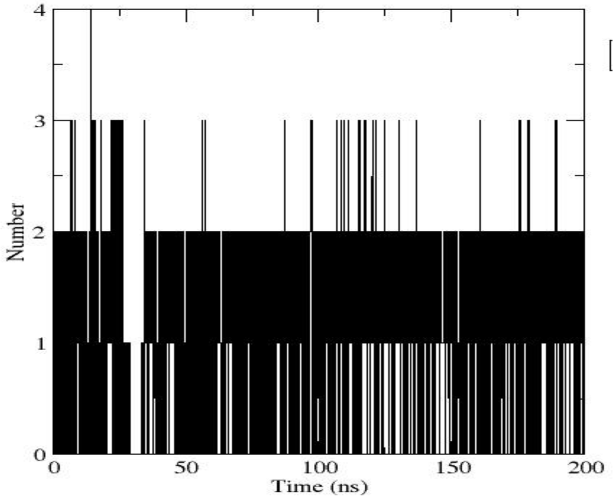
Number
0
1
2
3
4
Time (ns)
0
50
100
150
200

C

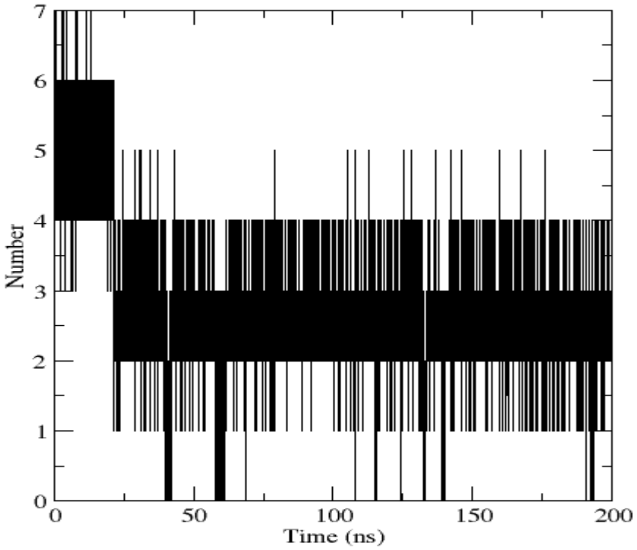
Number
0
1
2
3
4
5
6
7
Time (ns)
0
50
100
150
200

D

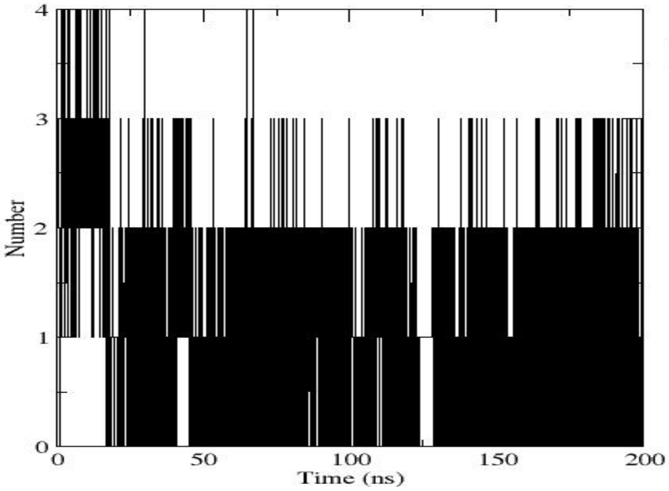
Number
0
1
2
3
4
Time (ns)
0
50
100
150
200

E

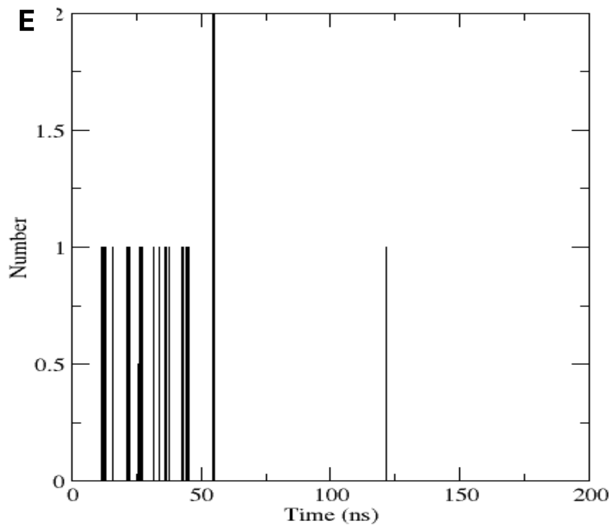

S4 Fig: Hydrogen bond occupancy analysis, showing the persistence of hydrogen bonding. interactions between secondary metabolites and key receptor residues, over a 200 ns simulation. The figure indicate the stability and strength of the ligand-protein interactions. A) Ellagic acid, B) Pulcherralpin, C) Myricetin, D) Quercetin, E) Lupeole Acetate.
