# Supplementary material for: In silico identification, high yielding isolation and in vitro validation of 6β-cinnamoyl-7β -hydroxyvouacapen – 5α - ol as a Wnt/β-catenin pathway targeted anti-cancer secondary metabolite of Caesalpinia pulcherrima
Source: PLoS One. 2025 Nov 3;20(11):e0334238. doi: 10.1371/journal.pone.0334238 (PMC12582477; doi:10.1371/journal.pone.0334238)
Supplement: S5 Fig — The spectrum confirms the presence of carbon signals corresponding to the compound’s structural framework. (PDF) [file pone.0334238.s008.pdf]

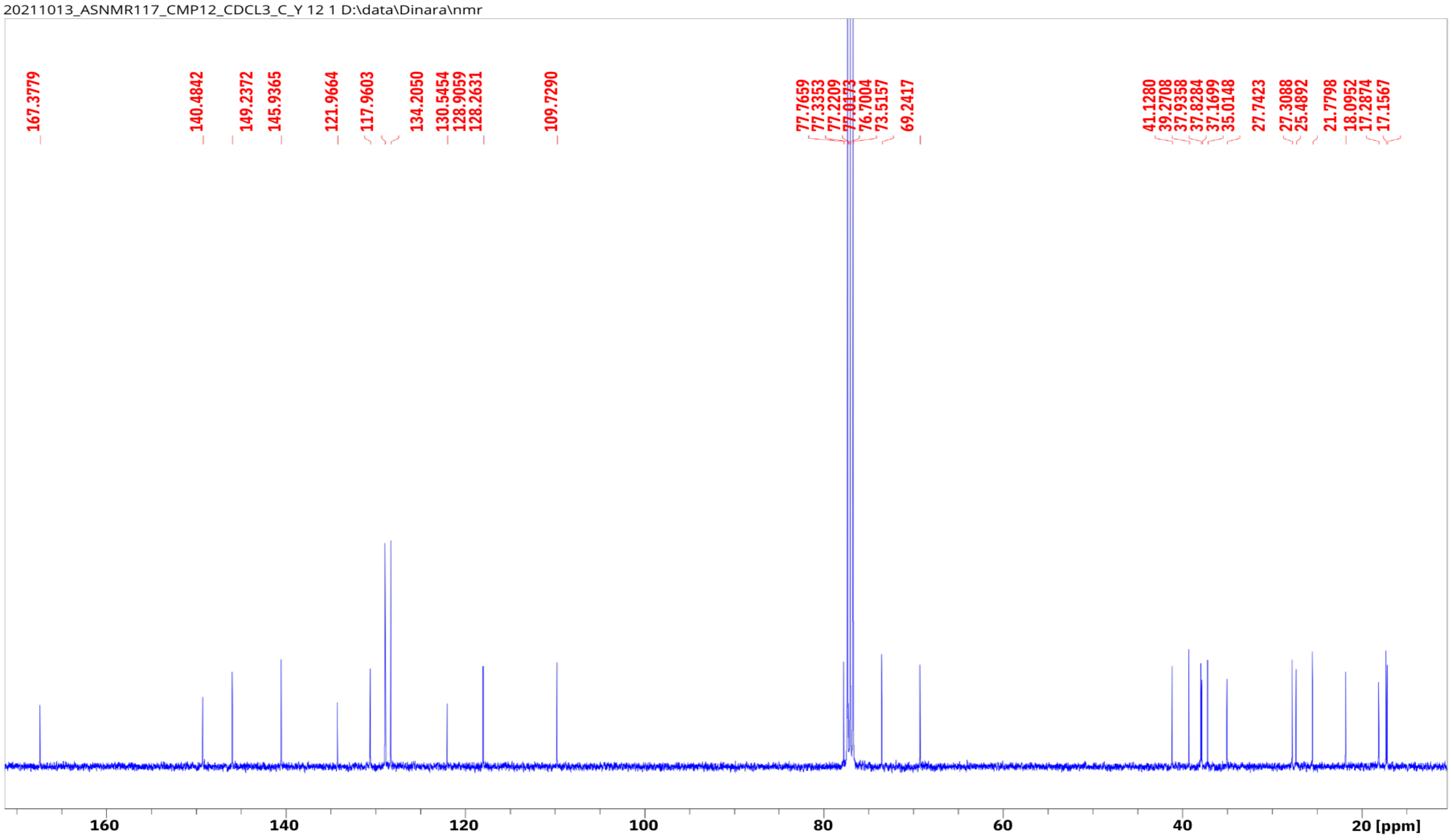

S5 Fig. The ^{13}C NMR spectrum of the compound 6βCHV. The spectrum confirms the presence of carbon signals corresponding to the compound's structural framework.
