# Supplementary material for: In silico identification, high yielding isolation and in vitro validation of 6β-cinnamoyl-7β -hydroxyvouacapen – 5α - ol as a Wnt/β-catenin pathway targeted anti-cancer secondary metabolite of Caesalpinia pulcherrima
Source: PLoS One. 2025 Nov 3;20(11):e0334238. doi: 10.1371/journal.pone.0334238 (PMC12582477; doi:10.1371/journal.pone.0334238)
Supplement: S6 Fig — This comparison confirms the similarity in Rf values and UV- active components between the two samples. (PDF) [file pone.0334238.s009.pdf]

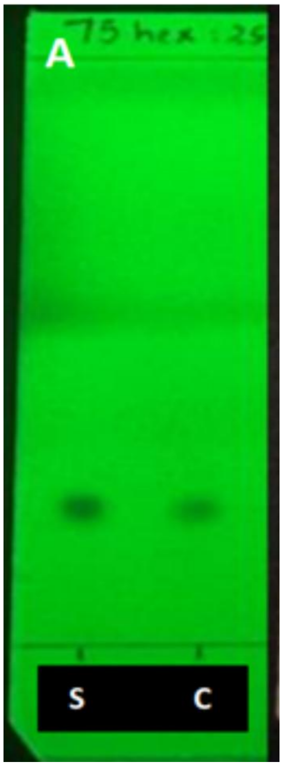

S6 Fig. TLC profile of isolated and recrystallized pure 6βCHV (C) and previously isolated 6βCHV (Erharuyi et al., 2016) (S) under (A) short UV (254 nm) wavelength. This comparison confirms the similarity in Rf values and UV- active components between the two samples.
