# Supplementary material for: In silico identification, high yielding isolation and in vitro validation of 6β-cinnamoyl-7β -hydroxyvouacapen – 5α - ol as a Wnt/β-catenin pathway targeted anti-cancer secondary metabolite of Caesalpinia pulcherrima
Source: PLoS One. 2025 Nov 3;20(11):e0334238. doi: 10.1371/journal.pone.0334238 (PMC12582477; doi:10.1371/journal.pone.0334238)
Supplement: S7 Fig — (PDF) [file pone.0334238.s010.pdf]

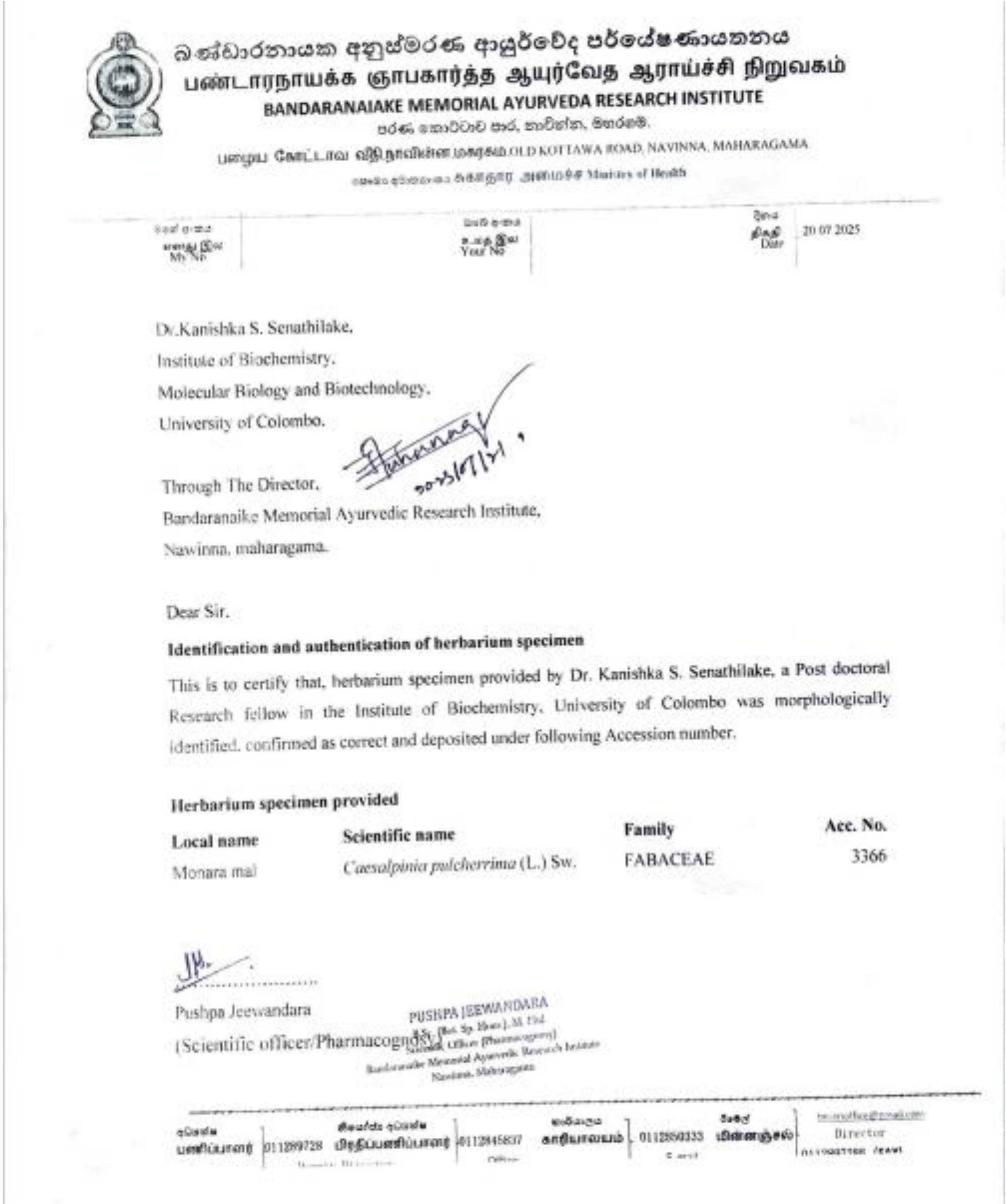

බණ්ඩාරනායක අනුස්මරණ ආයුර්වේද පර්යේෂණායතනය
பண்டாரநாயக்க ஞாபகார்த்த ஆயுர்வேத ஆராய்ச்சி நிறுவகம்
BANDARANAIAKE MEMORIAL AYURVEDA RESEARCH INSTITUTE
[illegible]
[illegible] OLD KOTTAWA ROAD, NAVINNA, MAHARAGAMA
[illegible] Ministry of Health

My No | Your No | Date 20 07 2025

Dr.Kanishka S. Senathilake,
Institute of Biochemistry,
Molecular Biology and Biotechnology,
University of Colombo.

Through The Director,
Bandaranaike Memorial Ayurvedic Research Institute,
Nawinna, maharagama.

Dear Sir,

Identification and authentication of herbarium specimen

This is to certify that, herbarium specimen provided by Dr. Kanishka S. Senathilake, a Post doctoral Research fellow in the Institute of Biochemistry, University of Colombo was morphologically identified, confirmed as correct and deposited under following Accession number.

Herbarium specimen provided

**Local name**	**Scientific name**	**Family**	**Acc. No.**
Monara mal	*Caesalpinia pulcherrima* (L.) Sw.	FABACEAE	3366

....
Pushpa Jeewandara
(Scientific officer/Pharmacognosy)

PUSHPA JEEWANDARA
[illegible]
Bandaranaike Memorial Ayurvedic Research Institute
Navinna, Maharagama

பணிப்பாளர் 0112899728 | பிரதிப்பணிப்பாளர் 0112845837 | காரியாலயம் 0112850333 | மின்னஞ்சல் [illegible] Director [illegible]

S7 Fig. Authentication letter for the plant specimen used in the study.
