# Supplementary material for: In silico identification, high yielding isolation and in vitro validation of 6β-cinnamoyl-7β -hydroxyvouacapen – 5α - ol as a Wnt/β-catenin pathway targeted anti-cancer secondary metabolite of Caesalpinia pulcherrima
Source: PLoS One. 2025 Nov 3;20(11):e0334238. doi: 10.1371/journal.pone.0334238 (PMC12582477; doi:10.1371/journal.pone.0334238)
Supplement: Attachment — Submitted filename: Response to reviwers.docx [file pone.0334238.s012.docx]

|  |  | Comment | Response |
| --- | --- | --- | --- |
| Journal Requirements | 1 | Please ensure that your manuscript meets PLOS ONE's style requirements, including those for file naming. | We have carefully revised our submission to ensure full compliance with PLOS ONE’s style requirements, and all figure, table, and supplementary files have been renamed and reformatted according to the journal’s guidelines. |
|  | 2 | In your Methods section, please provide additional information regarding the permits you obtained for the work. Please ensure you have included the full name of the authority that approved the field site access and, if no permits were required, a brief statement explaining why. | We have revised the Methods section (2.2.1) to include details on field site access and permits. (Line no. 145-148) |
|  | 3 | Please note that PLOS ONE has specific guidelines on code sharing for submissions in which author-generated code underpins the findings in the manuscript. In these cases, we expect all author-generated code to be made available without restrictions upon publication of the work. | In our study, all computational analyses (e.g., molecular dynamics and binding free energy calculations) were performed using publicly available software (GROMACS) and standard protocols. No novel or custom author-generated code was developed. To ensure clarity, we have provided the links in the supplementary document (table S1). |
|  | 4 | When completing the data availability statement of the submission form, you indicated that you will make your data available on acceptance. We strongly recommend all authors decide on a data sharing plan before acceptance, as the process can be lengthy and hold up publication timelines. Please note that, though access restrictions are acceptable now, your entire data will need to be made freely accessible if your manuscript is accepted for publication. This policy applies to all data except where public deposition would breach compliance with the protocol approved by your research ethics board. If you are unable to adhere to our open data policy, please kindly revise your statement to explain your reasoning and we will seek the editor's input on an exemption. Please be assured that, once you have provided your new statement, the assessment of your exemption will not hold up the peer review process. | Regarding the data availability policy. We have revised our Data Availability Statement as follow “All relevant data are within the manuscript and its Supporting Information files” in the cover letter. |
|  | 5 | Please state what role the funders took in the study. If the funders had no role, please state: "The funders had no role in study design, data collection and analysis, decision to publish, or preparation of the manuscript." If this statement is not correct you must amend it as needed. Please include this amended Role of Funder statement in your cover letter; we will change the online submission form on your behalf. | We have included the following amended statement in the cover letter as requested:  Role of Funders Statement: *“The funders had no role in study design, data collection and analysis, decision to publish, or preparation of the manuscript.”* |
|  | 6 | We note that you have provided funding information that is not currently declared in your Funding Statement. However, funding information should not appear in the Acknowledgments section or other areas of your manuscript. We will only publish funding information present in the Funding Statement section of the online submission form. Please remove any funding-related text from the manuscript and let us know how you would like to update your Funding Statement. Currently, your Funding Statement reads as follows: National Science Foundation, Sri Lanka  NSF/RPHS/2016-C07  Please include your amended statements within your cover letter; we will change the online submission form on your behalf. | We have removed the funding related text from the Acknowledgments section of the manuscript as requested. The update funding statement is stated in the cover letter. |
|  | 7 | We note you have included a table to which you do not refer in the text of your manuscript. Please ensure that you refer to Table 3 in your text; if accepted, production will need this reference to link the reader to the Table. | We respectfully note that Table 3 is already referred to in the Results section of the manuscript (Line no.287). |
|  | 8 | [Please include captions for your Supporting Information files at the end of your manuscript, and update any in-text citations to match accordingly. Please see our Supporting Information guidelines for more information: http://journals.plos.org/plosone/s/supporting-information.](http://track.editorialmanager.com/CL0/http:%2F%2Fjournals.plos.org%2Fplosone%2Fs%2Fsupporting-information./1/010f0197b03df672-3b73bca1-daca-45a1-b5f5-44fca98e5188-000000/3AHRG-ng_nRO2T8jIsYfpMFX7AUCWNGSfXi1bTvidos=217) | Captions for all Supporting Information files have now been included at the end of the revised manuscript, and the in-text citations have been updated to match the required PLOS ONE Supporting Information guidelines. |
| Additional Editor Comments |  | Broader safety profiling and dose-dependent comparative analyses are needed to assess therapeutic index more robustly. | We acknowledge the need for more extensive safety profiling. We have now included a discussion of this limitation and emphasized the need for future *in vivo* toxicological studies to better assess the therapeutic index (line 440-442). |
|  |  | Inclusion of additional pathway markers or proteomics could validate the proposed mechanism more thoroughly. | We agree that expanding mechanistic insights via additional pathway markers or proteomic profiling would enhance the strength of the findings. Due to resource constraints, these analyses were beyond the current study’s scope. However, we have now acknowledged this limitation in the discussion and proposed it as a critical next step in future work.  Line 451-455 |
|  |  | Clarify dose-response relationships and offer explanation for inter-cell line sensitivity differences. | **Already indicated content -** *In vitro* anti-proliferative activity assays revealed that 6βCHV exhibited significant *in vitro* anti-cancer activity, particularly against highly Wnt-dependent cancer cell lines AGS, HepG2, and SKOV3 (line 428 -439).  **New content added** - The observed inter-cell line differences in IC₅₀ and selectivity likely reflect variable dependence on Wnt/β-catenin signaling among these cancer types.  Dose dependent reduction of the cell viability was observed for all cell lines tested. This confirms that the decrease in cell viability is caused by the compound 6βCHV (line 432-435) |
|  |  | Reframe the novelty claim to reflect refinement or extension rather than complete originality. | We have revised the conclusion section to more accurately position the study as a refinement and extension of existing knowledge on diterpenoid compounds and Wnt pathway inhibitors, specifically focusing on the natural product 6βCHV. (Line 475-479) |
|  |  | Thorough professional language editing is required. Key data from supplements should be incorporated into the main manuscript figures or results section to aid clarity. | The manuscript has undergone thorough professional language editing to improve clarity, readability, and scientific presentation. Additionally, key data previously included in the supplementary material, have been incorporated into the main figures and Results section to make the findings more accessible and the manuscript more self-contained. |
| Reviewer 2 |  | The manuscript has potential. The authors have a clear therapeutic rationale and unmet-need focus. However, it needs major revisions to make it technically sound, address the lack of information about the study's statistical rigor and general language/presentation. | For the caspase activity assay, one-way analysis of variance (ANOVA) with Dunnet’s post-test was used to determine the significance difference of the response between doses. Results of RT-qPCR was analysed using Student’s t-test of the replicate 2^ (- Delta Ct) values for each gene in the control and treatment groups.  This was indicated in the statistical analysis section (Section 2.6). (line 242-245).  We have performed thorough professional language editing to improve readability, scientific clarity, and presentation throughout the manuscript. |
|  |  | The study is executed at the in-silico and in-vitro levels, so the main data do justify the narrow conclusion that 6β-Cinnamoyl-7β-hydroxyvouacapen-5α-ol (6βCHV) is a cytotoxic natural product that can down-regulate Wnt/β-catenin read-outs in cultured cancer and stem-like cells.  However, several methodological gaps and missing controls mean the evidence does not yet fully support the broader claims that 6βCHV “exerts its anticancer activity … by down-regulating the Wnt/β-catenin pathway” or that it is “a promising CSC-targeted therapeutic”. | We agree that our data support the specific, narrow conclusion that 6βCHV exhibits cytotoxicity and modulates Wnt/β-catenin read-outs in vitro (line 476-480).  Clarified methodological limitations and acknowledged missing controls in the Discussion, including the need for protein-level validation, broader mechanistic studies, and in vivo efficacy assessments, and Revised statements. Highlighted the need for further studies to establish CSC-targeted therapeutic potential in future work. |
|  |  | The manuscript signals significance with asterisks in the caspase-3/7 and qPCR figures (e.g. “**P < 0.001, ****P < 0.0001”) , but provides no information on: which statistical test was applied, whether data distribution and variance assumptions were checked, how many biological replicates (independent experiments) were analysed – only triplicate wells are mentioned, which are technical replicates ,whether any correction for multiple comparisons was used, exact P-values and confidence intervals. | We have revised the manuscript to provide full statistical details and incorporated in the Methods, Results, and figure legends to ensure full transparency and adherence to statistical reporting standards.  Figure 4 line 365-368  Figure 5 line 379-382  Figure 6 line 384-386 |
|  |  | Several tables and the IC₅₀ screens list point values without SD/SE or 95 % CI, preventing readers from judging variability or overlap between groups. Selectivity indices are reported without propagation of error, so their statistical meaning is unclear. Until these points are addressed, the statistical analysis cannot be considered appropriate or rigorous. | To address these concerns, we have revised the manuscript to ensure that the statistical analysis is now robust, interpretable, and consistent with reporting standards. |
|  |  | Regarding the presentation of the manuscript, I noticed multiple typos like "fare comparison of anti-proliferative activity" which should read “fair comparison”; numerous missing spaces and hyphens throughout; very long sentences spanning 4–5 lines (specially in the Discussion section). | The manuscript has undergone thorough professional language editing to correct typographical errors, improve spacing and hyphenation, and enhance readability. Long sentences, particularly in the Discussion section, have been restructured for clarity and conciseness while preserving scientific meaning. |
